# Eph receptor B6 shapes a cold immune microenvironment, inhibiting anti-cancer immunity and immunotherapy response in bladder cancer

**DOI:** 10.3389/fonc.2023.1175183

**Published:** 2023-08-09

**Authors:** Xiaolong Jia, Dongxu Zhang, Cheng Zhou, Zejun Yan, Zhaohui Jiang, Liping Xie, Junhui Jiang

**Affiliations:** ^1^ Department of Urology, First Affiliated Hospital, School of Medicine, Zhejiang University, Hangzhou, China; ^2^ Department of Urology, Ningbo First Hospital, Ningbo Hospital of Zhejiang University, Ningbo, China

**Keywords:** EPHB6, bladder cancer, immunomodulator, tumor microenvironment, immunotherapy, molecular subtype, prognostic signature

## Abstract

**Background:**

The role of Eph receptors and related ephrin (EFN) ligands (as the largest family of transmembrane-bound RTKs) in immunomodulation in many types of cancer, especially bladder cancer (BLCA), is scarcely known.

**Methods:**

A pan-cancer dataset was retrieved from The Cancer Genome Atlas (TCGA) to explore the relation between Eph receptor/EFN ligand family genes and immunomodulators and tumor-infiltrated immune cells (TIICs). Local BLCA, GSE32894, and GSE31684 cohorts were applied to validate. The IMvigor210 cohort was employed to explore the relationship between EPHB6 and immunotherapy response. Moreover, association between EPHB6 and molecular subtype was investigated to explore potential therapeutic strategies. Immunohistochemical staining of CD8 and CD68 was performed to validate the correlation between EPHB6 and TIICs.

**Results:**

The pan-cancer analysis revealed variations in the immunological effects of Eph receptor/EFN ligand family genes across different types of cancer. EPHB6 expression negatively correlated with the expression of the majority of immunomodulators (including HLA and immune checkpoints), and CD8 T cells and macrophages in both the TCGA-BLCA and validation BLCA cohorts, shaping a cold immune microenvironment with inhibited immunity. In the IMvigor210 cohort, patients with high-EPHB6 highly correlated with a non-inflamed, low PD-L1 expression immune phenotype, and correspondingly, with less responders to immunotherapy. The high-EPHB6 group, enriched with the basal subtype, presented significantly fewer *TP53* and more *FGFR3* genomic alterations. Finally, a novel EPHB6-related Genes signature, with reliable and robust ability in prognosis prediction, was constructed.

**Conclusions:**

This study comprehensively investigated the immunological effects of Eph receptor/EFN ligand family genes pan-cancer, and specially identified the immunosuppressive role of EPHB6 in BLCA. Furthermore, EPHB6 may predict the molecular subtype and prognosis of BLCA, and serve as a novel therapeutic target to improve the sensitivity of immunotherapy.

## 1 Introduction

Since the discovery of the first EphA1 in 1987, a growing body of evidence has revealed that Eph receptors and their ephrin (EFN) ligands are involved in various cell processes, including cell communication, proliferation and migration, and tissue homeostasis (1). Eph receptors, the largest family of transmembrane-bound receptor tyrosine kinases (RTKs), play crucial roles in angiogenesis, lymphangiogenesis, carcinogenesis, and metastasis across various types of cancer (2, 3). Based on sequence homology analysis, Eph receptors can be categorized into two classes: nine Eph A and five Eph B members, which differ primarily in the EFN ligand binding site. This difference is what determines the binding specificity between Eph receptors and EFNs (4).. Eph A and B receptors promiscuously bind to these five EFN A and three EFN B ligand members, respectively; however, cross interactions do occur between groups (5, 6). Eph receptors were found to signal through cross-talk with RTKs and other factors, that is, ligand-independent signaling (7).

Recently, the role of Eph receptors and EFN ligands in the immune system has been garnering attention, and Eph receptors especially have great potential to become immunotherapeutic targets. In addition to the ubiquitous expression in tumor cells, Eph receptors and EFN ligands are also widely expressed in immune cell subsets, such as monocytes, platelets, macrophages, dendritic cells, B cells, and T cells (8). EphA3, the first receptor found as a tumor-associated antigen, can be recognized by a CD4+ T cell clone in melanoma and this process stimulates selective immunoreactivity (9). EphA2-derived epitopes can induce the elevated immunoreactivity of CD4+ and CD8+ T cells in the EphA2-positive renal cell carcinoma (10), and dendritic cell vaccination with EphA2 peptides has been applied in the clinical trial of glioblastoma multiforme (11). Furthermore, several studies have revealed that the expression of Eph receptors in tumor or tumor-infiltrating immune cells is particularly with immunosuppressive response (8). Intrinsic EphA2 expression in pancreatic cancer cells correlated with the suppression of immune response because it retains the exclusion of T cells and causes a low infiltrating level of T cells; whereas, the knockout of EphA2 gene can reverse T cell exclusion, increase the abundance of CD4+ and CD8+ T cells, and improve the sensitivity to immunotherapy (12). EphA10 expression in breast cancer cells positively correlated with the expression of PD-L1 (13), indicating that more cancer cells can escape immune surveillance. Eph receptors have also been identified in many tumor infiltrating cells; for example, EphA3 is extensively expressed in stromal fibroblasts in many solid tumors, which may promote tumor progression and invasiveness, and also inhibit the anti-cancer immunity (14). Additionally, the expression of EFN ligands on monocytes and macrophages is closely associated with inflammation, and EFNB1-3 ligands may affect T cell receptor-mediated signaling and T cell co-stimulation (15).

BLCA is a significant genitourinary disease, with approximately 573,278 new cases and over 212,536 related deaths reported annually (16). Due to the frequent recurrence of BLCA in patients after transurethral resection of bladder tumors and the need for repeat surgeries, it is the most expensive cancer to treat on a per-patient basis (17). Currently, immune checkpoint inhibitors (ICIs) targeted at PD-1 or PD-L1 are becoming a novel and effective treatment option for BLCA, with durable antitumor efficacy. However, only about one-fifth of unselected patients respond to ICIs, and three prospective trials have shown that ICIs do not improve overall survival (OS) compared to chemotherapy alone (18–20). The limitations in improving the overall response rate for ICIs in BLCA therapy mainly stem from the individual heterogeneity in genetic and tumor immune microenvironment, and thus, the lack of robust predictive biomarkers to precisely select which patients will or will not benefit from the therapy (21–23). Therefore, it is necessary to identify novel biomarkers that can accurately predict immunotherapy response in BLCA patients. Eph receptors and EFN ligands prevalently correlated with the activation or suppression of innate and adaptive immune response in different types of cancer. Remarkably, patients with metastatic BLCA treated with combination therapy of EFNB2 inhibition and pembrolizumab had better OS and improved treatment responses than those receiving anti-PD-1/PD-L1 monotherapy (24). While the inhibition of PD-1/PD-L1 and Ephrin-related pathways has demonstrated promising outcomes in preclinical models (25), the expression pattern of Eph receptors and EFN ligands in most tumors, particularly BLCA, remains unclear, and their immunological roles are not well understood.

In this study, a pan-cancer analysis was initially performed to reveal the relationship between the Eph receptors and EFN ligands expression and immunoregulatory factors and identified inactive kinase EPHB6 which shaped a cold immune microenvironment and promoted the immune escape in BLCA. Previous studies have suggested that EPHB6 may have both oncogene (26) and tumor suppressor (27, 28) roles in different types of cancer. However, its specific function in BLCA remains unknown. Thereafter, the role of EPHB6 in the immune microenvironment, anti-cancer immunity, and as correlate of immunotherapy response of BLCA was investigated. Briefly, our study elucidated the expression patterns of Eph receptors and EFN ligands pan-cancer, and identified a novel biomarker of EPHB6 as a potential effective therapeutic target to improve the immunotherapy response of BLCA.

## 2 Materials and methods

### 2.1 Data acquisition and preprocessing

The transcriptomic data, mutational profile, copy number variant (CNV), methylation, and related clinical information of pan-cancer cohort, including 32 types of cancer, were retrieved from The Cancer Genome Atlas (TCGA). RNA-seq data were log2 (x+0.001) transformed, whereas TMB level was calculated using VarScan2 package. Based on the pan-cancer cohort, 406 patients with BLCA (all belonged to the muscle-invasive BLCA subtype, namely MIBC) were filtered and constituted as the TCGA-BLCA cohort. In addition, four other independent datasets of GSE32548, GSE13507, GSE188715, and GSE32894 from the Gene Expression Omnibus (GEO) were downloaded to verify the novel constructed prognostic signature.

### 2.2 RNA sequencing of cancer vs. normal tissues in the local BLCA cohort

Twenty-eight MIBC patients were enrolled in this study, and we have received their written informed consent. This study was approved by the Ethics Committee of Ningbo First Hospital. Tumor and matched normal tissues were collected from the patients in the local BLCA cohort after the surgical resection. A FastPure^®^ Cell/Tissue Total RNA Isolation Kit V2 (Vazyme, Jiangsu, China) was used to extract total RNA, which was quantified and qualified using the Qubit (ThermoFisher Scientific, MA, the United States) and Agilent 2100 bioanalyzer (Agilent Technologies, CA, United States), respectively. The NEBNext^®^ Ultra™ RNA Library Prep Kit (NEB, MA, US) was subsequently used for the construction of RNA library. The prepared RNA library was finally sequenced on the Illumina Novaseq-6000 machine (Illumina, MA, US).

### 2.3 Immune-related features and anti-cancer immunity in BLCA

In this study, several indices including immunomodulator gene expression, tumor immune cell infiltration, cancer immunity cycle, and inhibitory immune checkpoints-related gene expression were employed for the evaluation of the immune-related features, anti-cancer immunity, and microenvironmental status in BLCA. A total of 122 immunomodulators (including chemokines, paired receptors, MHC molecules, and immunostimulators), which have been reported previously, were collected to estimate the immunomodulation of TME in BLCA (29). The cancer immunity cycle was reviewed to represent the anti-cancer immunity, and the following contained seven steps. Step 1: release of cancer cell antigens, step 2: cancer antigen presentation, step 3: priming and activation, step 4: trafficking of immune cells to cancer cells, step 5: immune cell infiltration into tumor, step 6: recognition of cancer cells by T cell, and step 7: killing of cancer cell (30). These steps were performed using a single sample gene set enrichment analysis (ssGSEA), based on the transcriptomic data of each sample (31). To decrease the errors, several algorithms, including TIMER, CIBERSORT, QUANTISEQ, MCPCOUNTER, XCELL, and EPIC algorithms, were performed to assess the infiltrating levels of tumor immune cells (mainly CD8+, Macrophage, Dendritic cells, Natural Killer (NK) cells, and Th1 cells). The ESTIMATE algorithm was also employed to evaluate the immunological status of TME in BLCA. Furthermore, the profile of inhibitory immune checkpoints was obtained from a study by Auslander (32). As described, a pan-cancer T cell inflamed score (an 18 genes signature (33),) associated with pre-existing cancer immunity, which could predict the response of immunotherapy.

### 2.4 IMvigor210 cohort defined three immune phenotypes in BLCA

The transcriptomic data and related clinical information of 348 patients with urothelial cancer (UC) in the IMvigor210 cohort were downloaded from: http://research-pub.gene.com/IMvigor210CoreBiologies/. These patients with UC reportedly received an immunotherapy *via* anti-PD-L1/PD-1 antibodies. Accordingly, the therapy response was defined similarly as the published criteria (34): CR: complete response, PR: partial response, SD: stable disease, and PD: progressive disease; and CR/PR and SD/PD were defined as the binary response groups. In the IMvigor210 cohort, BLCA samples were categorized into three immune phenotypes, namely deserted, excluded, and inflamed microenvironment, according to the distribution, status, and infiltrating level of CD8+ T cells. In addition, IC0, IC1, and IC2+ phenotypes were characterized as the lowest, medium, and the highest PD-1 expression, respectively. Thus, these known parameters were employed in this study to determine the role of EPHB6 during immunotherapy. Eventually, a majority of immunotherapy-predicted pathways were performed using the ssGSEA algorithm, and we further explored the association between EPHB6 and the activities of these immunotherapy-predicted pathways.

### 2.5 Molecular characterization underlying EPHB6

The Gene Ontology (GO), Kyoto Encyclopedia of Genes and Genomes (KEGG) pathway, and Hallmark pathway enrichment analysis in this study was conducted to explore the EPHB6-associated signaling pathways. First, the differentially expressed genes (DEGs; log2 (Fold change)>1.5; p<0.01) between EPHB6-high and -low expression groups were acquired. Then, a functional enrichment analysis of these DEGs was conducted using the fgsea package to calculate the enrichment score. In the GO enrichment analysis, three categories of BP were delineated: biological process (GOBP), CC: Cellular component (GOCC), and MF: molecular function (GOCC). In addition, the whole genome sequencing data in the TCGA-BLCA cohort were downloaded to visualize the overall mutational profile in BLCA and the differences in the genomic landscape between EPHB6-high and -low expression groups using maftools package. Genomic alteration enrichment analysis was performed to directly display the prevalent genes in different groups.

### 2.6 EPHB6, molecular subtype, and therapeutic relations

In addition to the TMB, microsatellite instability, and inhibitory immune checkpoints, several studies have revealed that molecular subtype may predict the response of some therapies in BLCA (35–38). In this study, several molecular subtyping strategies were used, including Consensus, CIT, Lund, MDA, TCGA, Baylor, and UNC subtypes. Accordingly, some pathways involved with urothelial differentiation, Ta pathway, cell cycle activity, mitochondria, basal differentiation, myofibroblasts, interferon (IFN) response, B and T lymphocytes, smooth muscle, and neuroendocrine differentiation were used for evaluation of each molecular subtype, and ssGSEA was conducted to calculate the enrichment score. The receiver operating characteristic (ROC) curve and the value of area under ROC curve (AUC) were used to evaluate the accuracy of EPHB6 in predicting the molecular subtype. As reported, *RB1* (39), *ATM* (40), *ERBB2* (41), *ERCC2* (42), and *FANCC* (43) alterations highly correlated with the response of adjuvant or neoadjuvant chemotherapy. In addition, the Drugbank database was used to predict the potential target drugs against BLCA.

### 2.7 Immunohistochemical analysis of EPHB6 and correlation with TME

We collected specimens from a local cohort of 21 BLCA patients to analyze the expression level of EPHB6 and its correlation with TME feature. These patients were classified into three groups: NMIBC-low grade (n=7), NMIBC-high grade (n=7), and MIBC-high grade (n=7). Tissue samples (4-µm-thick sections) were subjected to immunohistochemical staining for EPHB6, CD8, CD68 using EPHB6 (ab217542, 1:100, Abcam) CD8 (ab217344, 1:100 Abcam) and CD68 (ab955, 1:50, Abcam) antibodies, respectively. The immunohistochemical staining method was performed as described previously by Sun et al. (44). The staining score for EPHB6 was classified as high (staining in more than 50% of malignant cells), low (staining in less than 25% of malignant cells), or medium (staining in 25%-50% of malignant cells). The staining of CD8 and CD68 was only evaluated in intratumoral infiltrating leukocytes. The score was calculated independently by two pathologists from five high magnification (200) visual fields using CaseViewer2.2 (Thermo Fisher).

### 2.8 Construction of a EPHB6-related genes prognostic signature

Finally, the prognostic value of Eph receptor/EFN ligand family genes were explored *via* univariate Cox regression analysis. Notably, EPHB6 markedly correlated with the prognosis of BLCA, indicating that EPHB6 also can be a potential prognostic biomarker. To identify EPHB6-related differentially expressed genes (DEGs), we employed the limma R package to compare tumor vs. normal tissues and high- vs. low-EPHB6 expression groups in the TCGA-BLCA dataset. The criteria for determining differential DEGs were adjusted P-value < 0.01 and |log(fold change)|>1. We then used the VennDiagram R package to collect and analyze the overlapped genes. Based on the DEGs analysis, 35 prognosis-related genes were screened out for the construction of a EBGs prognostic signature. In this study, seven kinds of machine learning algorithms were applied, including RF, Ridge, stepwise Cox, Enet, Boost, multivariate Cox, and LASSO, of which two methods were randomly selected to discover the most robust EBGs signature, as the standard of the highest C-index in the training and four independent validation cohorts. The Kaplan–Meier curve analysis was conducted to compare the OS between high- and low- EBGs score groups. The ROC curve and corresponding AUC value were used to evaluate the reliability and robustness of EBGs signature. Moreover, the predictive performance of EBGs signature was further verified in four other independent cohorts: GSE32548, GSE13507, GSE188715, and GSE32894 from the GEO database.

### 2.9 Statistical analysis

In this study, all statistical analyses were conducted using the R studio. Correlation analyses between variables were performed using Pearson coefficients. Categorical variables analysis was conducted using chi-square and Fisher’s exact tests. In addition, Mann–Whitney U test was performed for continuous variables. In the Kaplan–Meier curve analysis, the log-rank test was performed. The data was considered statistically significant at p<0.05. All applied software and version information was provided in the **Supplementary Table 1**.

## 3 Results

### 3.1 Immunological correlation between Eph receptors and EFN ligands in pan-cancer cohort

**Figure 1:**
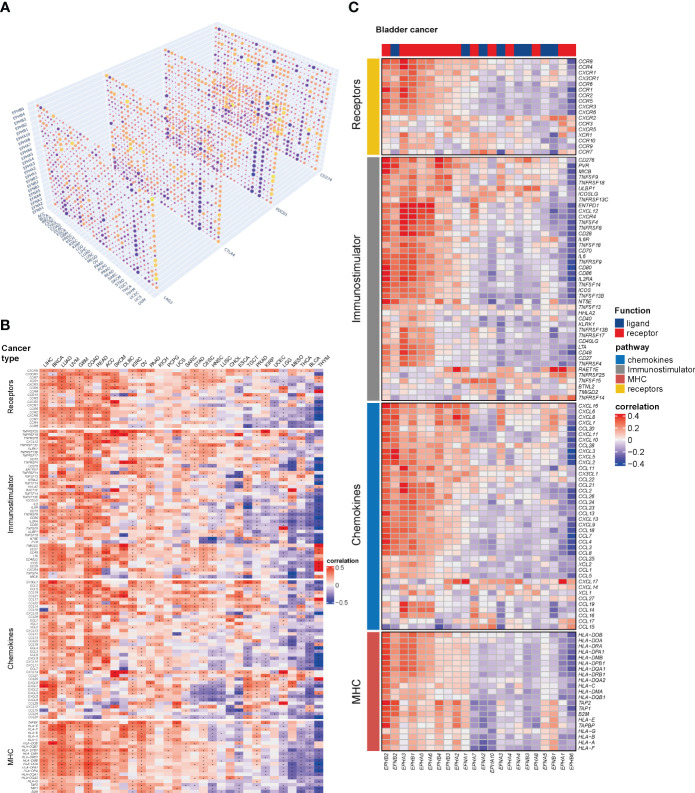
Immunological correlation between Eph receptors and EFN ligands in the pan-cancer cohort. **(A)** Correlation matrix showing the relationship between the expression levels of all the Eph receptors and EFN ligands and LAG3, CTLA4, PDCD1 and CD274 in TCGA pan-cancer cohorts. Dots labeled with yellow color indicated a negative correlation between genes and checkpoints in a tumor type; whereas, those with purple indicated a positive correlation. **(B)** Heatmap revealing the association between immunomodulatory genes and all the Eph receptors and EFN ligands in TCGA pan-cancer cohorts. **(C)** Correlation analysis showing the relationship between all the Eph receptors and EFN ligands and immunomodulatory genes in TCGA-bladder cancer (BLCA) cohort alone. *p<0.05.

The immunological correlation between Eph receptors and EFN ligands in the TCGA pan-cancer cohorts was investigated. For most types of cancer, a positive and significant association was noted between most of Eph receptors/EFN ligands and immunomodulatory genes at the transcriptomic level (**Supplementary Figure 1**). Furthermore, a notable heterogeneity was observed in the relationship between Eph receptors/EFN ligands and main checkpoints (CD274, PDCD1, CTLA4, and LAG3) in different types of cancer (**Figure 1A**). For the first time, it was identified that EPHB6, a previously recognized inactive kinase receptor, had a significantly relation with immunomodulatory genes expression in most types of cancer (**Figure 1B**). Consistent with the result exhibited in **Figure 1A**, EPHB6 expression was found to have a significantly negative correlation with immunomodulatory genes expression in mesonephric adenocarcinoma (MESO), thyroid carcinoma (THCA), thymoma (THYM), and BLCA. In BLCA, EPHB6 demonstrated a different immunological profile compared to other Eph receptor/EFN ligand family genes, which showed the most negative connection with the majority of immunomodulators (**Figure 1C**).

### 3.2 EPHB6 is related to the survival of patients with BLCA

**Figure 2:**
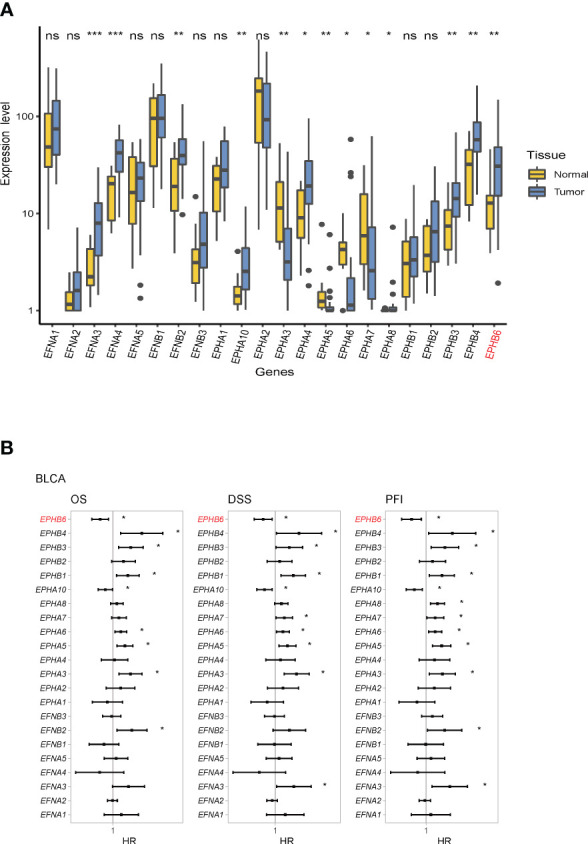
Expression and prognostic role of Eph receptors and EFN ligands in bladder cancer. **(A)** Difference in the mRNA expression level between bladder cancer tumor tissues and normal tissues in local bladder cancer cohort. **(B)** Forest plot for the association between Eph receptors and EFN ligands and survival by median cutoff in the TCGA-BLCA cohort; data were presented with HR and 95% CI. * p<0.05. OS, overall survival; DSS, disease-specific survival; PFI, progression-free interval; HR, hazard ratio. *p<0.05; **p<0.01; ***p<0.001; ns: non-significant.

In the local BLCA cohort, most Eph receptor genes (EphA3, EphA4, EphA5, EphA6, EphA7, EphA8, EphA10, EphB3, EphB4, and EPHB6) and several EFN ligand genes (EFNA3, EFNA4, and EFNB2) expressions were significantly dysregulated (p<0.05; **Figure 2A**). This result suggested that these Eph receptor/EFN ligand family genes may be also correlated with the prognosis of BLCA. Subsequently, univariate Cox regression analysis revealed that EPHB6 and EphA10 expressions were positively correlated with OS, DSS, and PFI; whereas, EphB4, EphB3, EphB1, EphA6, EphA5, and EphA3 expressions were negatively correlated with OS, DSS, and PFI (p<0.05; **Figure 2B**). Based on these features, the following experiments mainly focused on investigating the role of EPHB6 and tumor immunology in BLCA.

### 3.3 EPHB6 is associated with a cold immune microenvironment in BLCA

**Figure 3:**
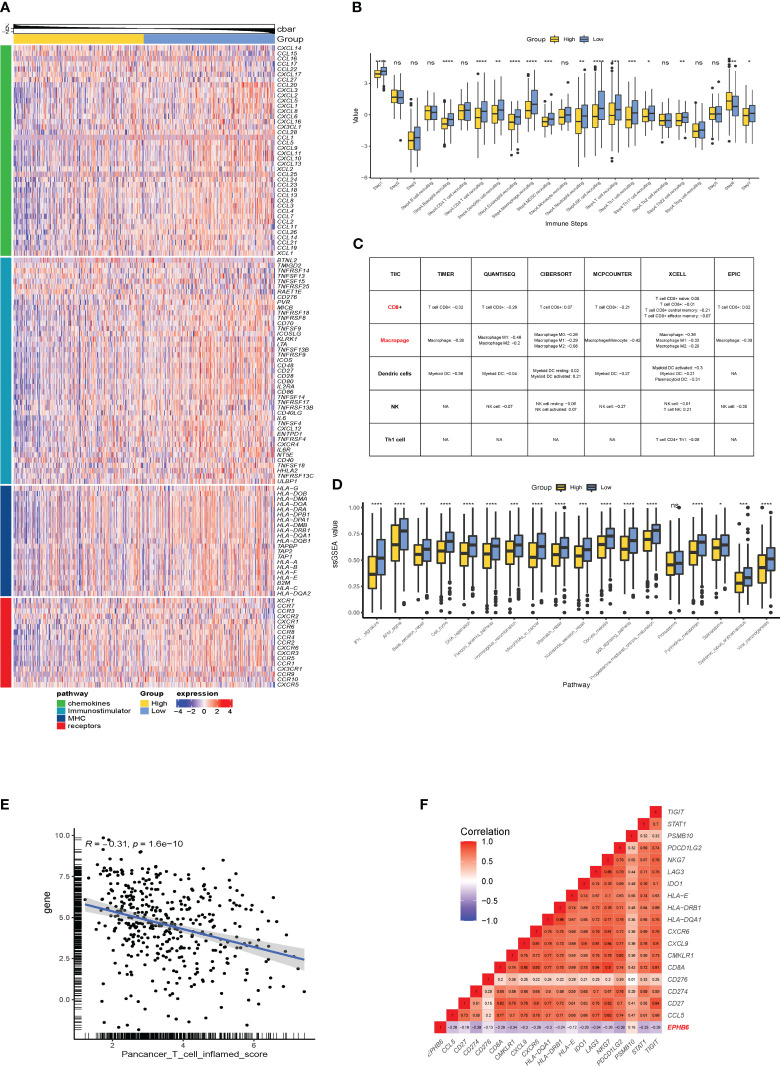
EPHB6 is associated with a cold immune microenvironment in bladder cancer. **(A)** Difference in the expression levels of immunomodulatory genes between the high- and low-EPHB6 groups in the TCGA-BLCA cohort. **(B)** Bar plots showing the difference in the activities of seven immune steps between the high- and low-EPHB6 groups in the TCGA-BLCA cohort. **(C)** Correlation between EPHB6 expression levels and tumor-infiltrated immune cells (TIICs) in bladder cancer. The levels of TIICs were analyzed by multiple algorithms, including TIMER, CIBERSOFT, QUANTISEQ, MCPCOUNTER, XCELL and EPIC. **(D)** Bar plots revealing the difference in the immune-related signal pathways between the high- and low-EPHB6 groups. **(E)** Correlation between EPHB6 expression level and T cell inflamed score. **(F)** Correlation between EPHB6 and each individual T cell inflamed signature-related gene expression. *p<0.05; **p<0.01; ***p<0.001; ****p<0.0001; ns, non-significant.

Subsequently, the immunological effects regulated by EPHB6 expression were deeply investigated in the TCGA-BLCA cohort. As demonstrated, the expressions of most chemokines and their paired receptors (*CXCL9*, *CXCL10*, *CXCL11*, *CXCL13*, *CCL3*, *CCL4*, *CCL8*, *CCR2*, *CCR3*, *CCR4*, *CCR5*, *CCR6*, *CCR8*, *CXCR3*, and *CXCR6*) were significantly lower in the EPHB6-high expression group when compared to those in the EPHB6-low expression group (p<0.05; **Figure 3A**). Most immunostimulators (including TNFSF4, TNFSF14, TNFRSF9, TNFSF13B, TNFRSF17, CD27, CD28, CD48, and CD80) were also downregulated in the EPHB6-high expression group. Furthermore, expressions of several MHC genes (HLA-DMA, HLA-DMB, HLA-DOA, HLA-DOB, HLA-DQA1, HLA-DQB1, HLA-DPA1, HLA-DPB1, HLA-A, HLA-B, HLA-E, and HLA-F) were negatively correlated with EPHB6 expression. In summary, EPHB6 may shape a cold immune microenvironment in BLCA. Correspondingly, EPHB6 expression negatively correlated with most steps engaged in cancer immunity cycle, including the step 1 of cancer cell antigen release and step 4 of basophil, CD8+ T cell, dendritic cell, eosinophil, macrophage, MDSC, neutrophil, NK cell, Th1 cell, Th17 cell, and Th22 cell recruiting (p<0.05; **Figure 3B**). However, step 6 activity of recognition of tumor cells by T cells was significantly downregulated in the EPHB6-low expression group (p<0.001), probably owing to the high level of inhibitory immune checkpoints stimulating immune escape. The step 7 activity of killing of cancer cells was significantly upregulated in the EPHB6-low expression group (p<0.05; **Figure 3B**). Notably, it was consistent that EPHB6 expression negatively correlated with macrophages, dendritic cells, NK cells, and CD8+ T cells infiltrating levels in different algorithms, including TIMER, CIBERSORT, QUANTISEQ, MCPCOUNTER, XCELL, and EPIC (**Figure 3C**). Similarly, the effector genes expression of these immune cells was downregulated in the EPHB6-high expression group (**Supplementary Figure 2A**). Furthermore, EPHB6 was found to have a negative relation with nearly all known inhibitory immune checkpoints at the transcriptomic level, including PD-L1, PD-1, CTLA4, LAG3, TIGHT, IDO1, and IDO2 (**Supplementary Figure 2B**). Notably, in the TCGA-BLCA cohort, EPHB6 expression was negatively correlated with the enrichment scores of most immunotherapy predicted pathways, such as IFN-γ signaling, DNA damage repair (DDR) signaling, cell cycle, and DNA replication related signaling (p<0.05; **Figure 3D**). As previously reported, T cell inflamed score is associated with immunotherapy response; therefore, the relation between EPHB6 expression and T cell inflamed score was evaluated, demonstrating that EPHB6 expression had a remarkably negative correlation with pan-cancer T cell inflamed score (R = -0.31, p<0.0001; **Figure 3E**). EPHB6 expression also negatively correlated with each individual T cell inflamed signature-related gene expression (**Figure 3F**).

### 3.4 Immunological role of EPHB6 in local BLCA cohort and other independent datasets

**Figure 4:**
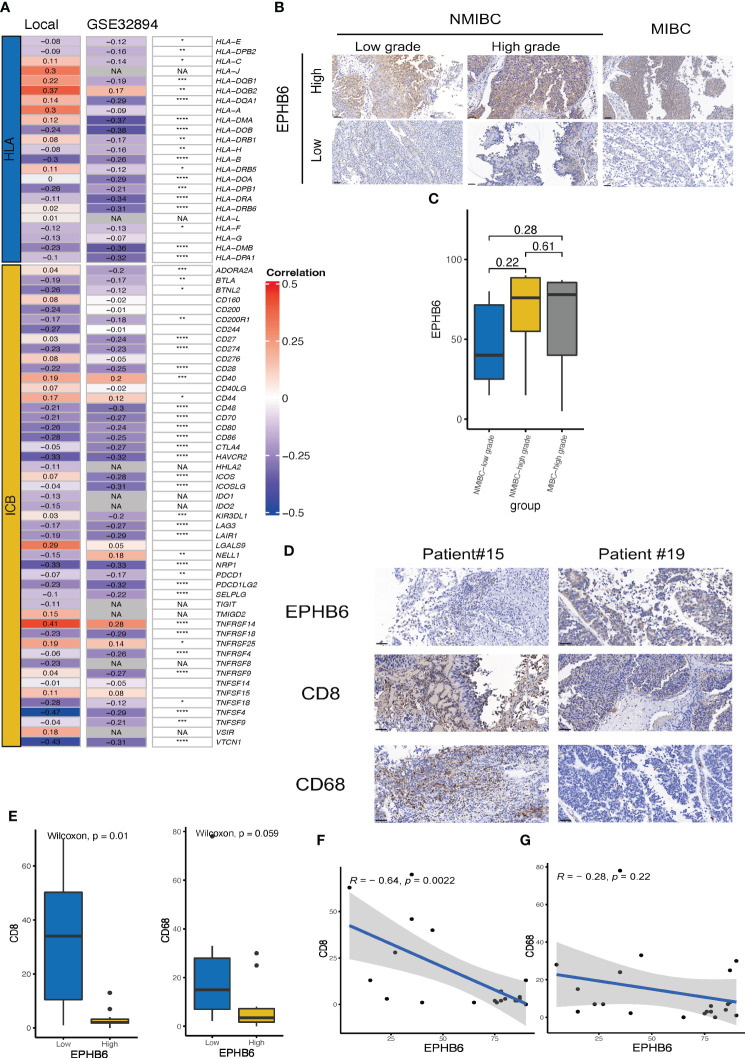
Validation of the association between EPHB6 and immunology feature in local and other bladder cancer datasets. **(A)** Correlation analysis of the mRNA expression levels of immunomodulatory genes (HLA and ICBs) and EPHB6 in local bladder cancer (BLCA) and GSE32894 datasets. **(B)** Immunohistochemical staining of EPHB6 in patients with NMIBC-low grade (left panel), NMIBC-high grade (middle panel), and MIBC (right panel) from local cohort. **(C)** Bar plot showing the difference in EPHB6 protein expression level in patients with NMIBC-low grade, NMIBC-high grade, and MIBC from local cohort. The images were captured at a magnification of 200, and a scale bar indicating 50 μm was included in the left bottom corner of each image. **(D)** Representative image of immunohistochemical staining of EPHB6, CD8 and CD68 in patients with high- and low-EPHB6 protein expression level. The images were captured at a magnification of 200, and a scale bar indicating 50 μm was included in each image’s left bottom corner. **(E)** Bar plot showing the difference in the CD8 and CD68 protein expression levels in patients with high- and low-EPHB6 protein expression level. Correlation analysis of the protein expression levels of EPHB6 and CD8 **(F)** or CD68 **(G)** in BLCA patients from local cohort. HLA, human leukocyte antigens; ICB, immune checkpoint; NMIBC, non-muscle invasive bladder cancer; MIBC, muscle invasive bladder cancer. *p<0.05; **p<0.01; ***p<0.001; ****p<0.0001; NA: not applicable.

In the local BLCA and GSE32894 cohorts, we observed a significant negative correlation between the expressions of most HLA-family members and immune checkpoints and the mRNA expression level of EPHB6 (**Figure 4A**). However, HLA-DQB2, CD40, CD44, and TNFRSF14 were exceptions to this correlation. These findings were further supported by similar results obtained in the GSE31684 cohort (**Supplementary Figure 3**), which confirmed that EPHB6 may contribute to the development of a cold immune microenvironment in BLCA. Considering the consistent negative correlation between EPHB6 mRNA expression level and the abundance of CD8 T cells and macrophages, we conducted immunochemical staining of EPHB6, CD8, and CD68 to validate this correlation. Firstly, we observed heterogeneity in the protein expression level of EPHB6 among BLCA patients. However, no significant difference in the protein expression level of EPHB6 was found among patients with NMIBC-low grade, NMIBC-high grade, and MIBC in the local cohort (**Figures 4B, C** **)**. Furthermore, there was a noticeable difference in the staining of CD8 and CD68 between BLCA patients with high and low EPHB6 expression levels (**Figures 4D, E** **)**. Subsequently, we found a significant negative correlation between EPHB6 and CD8 (r=-0.64, p<0.01, **Figure 4F**). Although the correlation between EPHB6 and CD68 was also negative (r=-0.22), this difference was not statistically significant in our protein analysis (**Figure 4G**). Nevertheless, the negative correlation between the protein expression profile of EPHB6 and CD8 or CD68 was consistent with the mRNA expression correlation observed in multiple datasets (**Supplementary Figure 4**).

### 3.5 EPHB6 is associated with immunotherapy response in BLCA

**Figure 5:**
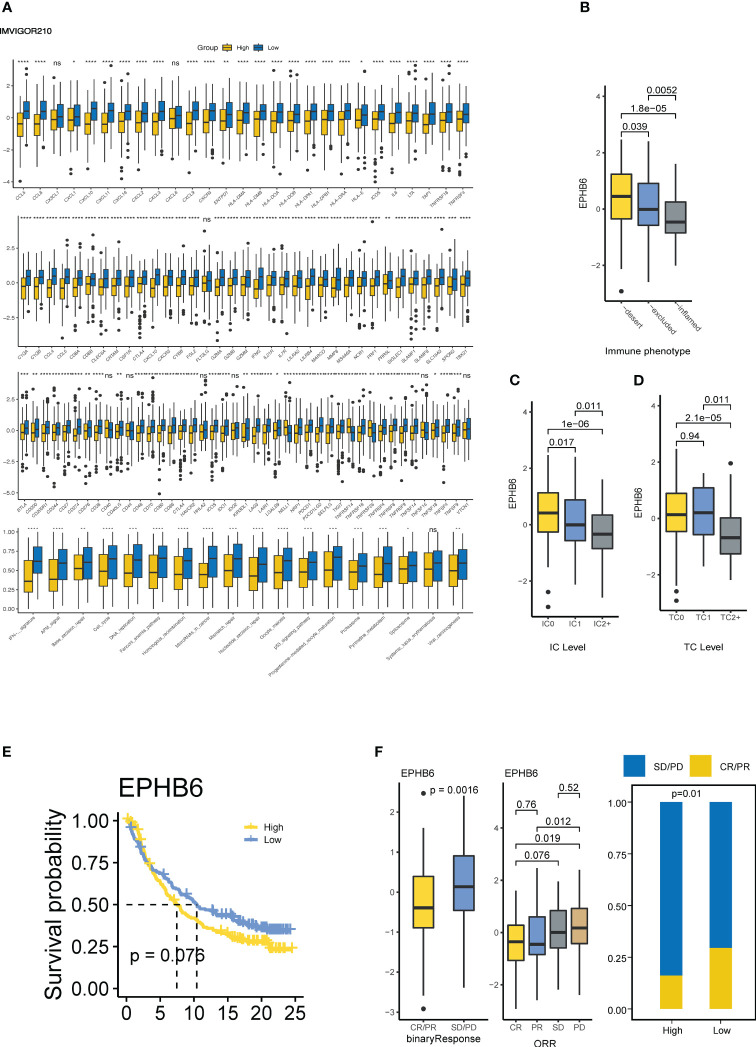
EPHB6 is associated with immunotherapy response in bladder cancer. **(A)** Difference in the expression levels of immunomodulatory genes between high- and low-EPHB6 groups in Imvigor210 cohort. Difference in the EPHB6 mRNA expression level between groups with various immune phenotypes **(B)**, PD-L1 expression in immune cells **(C)** PD-L1 expression in tumor cells **(D)**. **(E)** Kaplan-Meyer plot comparing patients with high- or low-EPHB6 expression levels treated with immunotherapy. **(F)** Difference in the EPHB6 mRNA expression level between responders (CR/PR) and non-responders (SD/PD) treated with immunotherapy. IC: PD-L1 immunohistochemistry levels in immune cells: IC0 (<1%); IC1(≥1% and <5%); IC2+ (≥5%). TC: PD-L1 immunohistochemistry levels in tumor cells TC0 (<1%); TC1 (≥1% and <5%); TC2+ (≥5%); IC0 (<1%); IC1 (≥1% and <5%); IC2+ (≥5%); CR, complete response; PR, partial response; SD, stable disease; PD, progressive disease. *p<0.05; **p<0.01; ***p<0.001; ****p<0.0001; ns, non-significant.

Furthermore, the immunological role of EPHB6 was evaluated in the IMvigor210 cohort. Patients with UC in the EPHB6-low expression group had a markedly higher level of immunomodulators, T cell inflamed signature-related genes, immune checkpoints, and immunotherapy predicted pathways which was consistent with the cold immune feature as previously mentioned (**Figure 5A**). Based on the classification of three immune phenotypes (deserted, excluded, and inflamed) in the IMvigor210 cohort, deserted and inflamed phenotype groups were found to have the highest and lowest EPHB6 expression, respectively (p<0.05; **Figure 5B**). According to the PD-1 expression status, higher PD-L1 expressed immune cells group (IC2+, with the highest PD-1 expression) had the lowest EPHB6 expression than that in the IC0 and IC1 phenotype groups; conversely, the highest EPHB6 expression was observed in the IC0 phenotype group (with the lowest PD-1 expression) when compared to the IC1 and IC2+ phenotype groups (p<0.05; **Figure 5C**). Moreover, TC2+ phenotype group (with the highest PD-L1 expression on tumor cells) also had the lowest EPHB6 expression (p<0.05); however, no significant difference was noted in EPHB6 expression between TC0 and TC1 phenotype groups (**Figure 5D**). Briefly, EPHB6 expression had a strongly negative correlation with the cold immune microenvironment in UC. Patients with high-EPHB6 in the IMvigor210 cohort had an inferior OS (**Figure 5E**). Remarkably, in the IMvigor210 cohort, patients with UC who showed a complete or partial response (CR/PR) to immunotherapy had the significantly downregulated expression of EPHB6, compared with those with a progressive or stable disease (PD/SD) (p<0.01; **Figure 5F**). Significantly higher proportion of responders to immunotherapy were present in the low-EPHB6 group. Notably, after both TP53 mutation and EPHB6 stratification, we found that TP53^mt^EPHB6^low^ group and TP53^mt^EPHB6^high^ had the highest and lowest proportion of responders, respectively (**Supplementary Figure 5**). Meanwhile, differences between high- and low-EPHB6 group were nearly insignificant when TP53 was not mutated.

### 3.6 Biological enrichment underlying EPHB6

**Figure 6:**
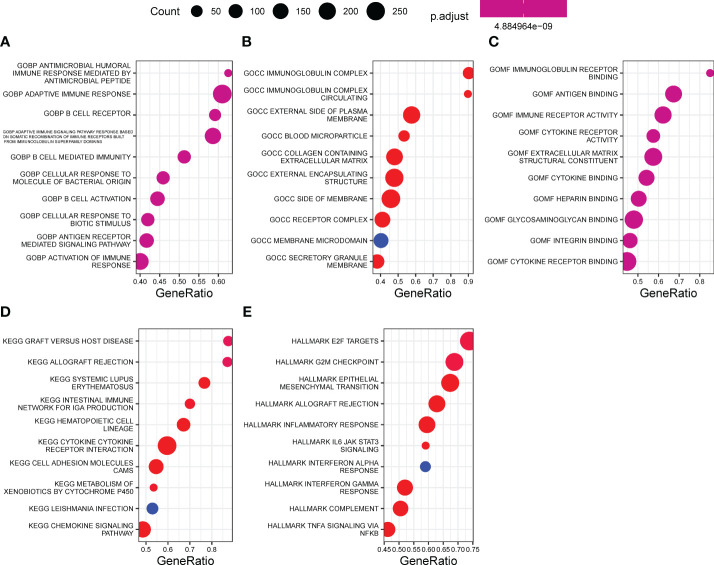
Biological enrichment underlying EPHB6. Function enrichment of EPHB6 expression related DEGs by GO **(A-C)**, KEGG **(D)** and hallmark **(E)**. DEG, differentially expressed genes; GOBP, gene ontology biological process; GOCC, gene ontology cellular component; GOMF, gene ontology molecular function; KEGG, kyoto encyclopedia of genes and genomes.

Underlying EPHB6 expression-related DEGs, GOBP of antimicrobial humoral immune response mediated by antimicrobial peptide, B cell mediated immunity, antigen receptor-mediated signaling pathway, and activation of immune response; GOCC of immunoglobulin complex, collagen-containing extracellular matrix, and receptor complex; and GOMF of immunoglobulin receptor-binding, antigen-binding, immune receptor activity, and cytokine receptor activity were significantly enriched (**Figures 6A-C**). KEGG pathway enrichment analysis further revealed that cytokine–cytokine receptor interaction, metabolism of xenobiotics by cytochrome P450, and chemokine signaling pathway were differentially regulated between the EPHB6-high and -low expression groups (p<0.0001; **Figure 6D**). Hallmark pathway enrichment analysis displayed the enrichment of E2F targets, G2M checkpoint, epithelial–mesenchymal transition, allograft rejection, inflammatory response, IL6-JAK-STAT3 signaling, IFN-α response, IFN-γ response, complement, and TNFα signaling *via* NFκB (p<0.0001; **Figure 6E**). Biological enrichment analysis verified that EPHB6 expression was markedly associated with the regulation of immune-related pathways.

### 3.7 Genomic characteristics associated with EPHB6

**Figure 7:**
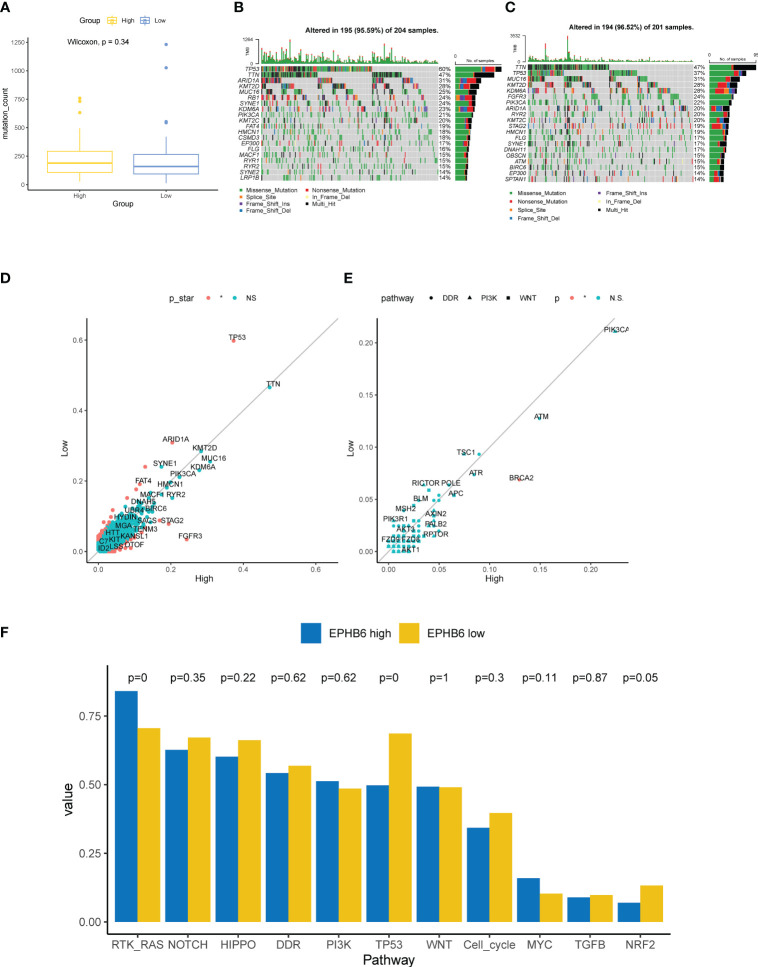
Genomic characteristics associated with EPHB6. **(A)** Difference in the mutation counts level between high- and low-EPHB6 group. Prevalent mutated genes in high- **(B)** and low-EPHB6 group **(C)**. **(D)** Difference in the prevalence of mutated genes between high- and low-EPHB6 group. Genes colored in red were those significantly differed (p<0.05). **(E)** Difference in the prevalence of genes involved in DDR, PI3K or WNT signaling pathways between high- and low-EPHB6 group. **(F)** Difference in the mutated signaling pathways between high- and low-EPHB6 group.

Underlying EPHB6 expression, we further deeply investigated the differences in genomic characteristics between EPHB6-high and -low expression groups. In spite of no statistical significance existing in the TMB level between EPHB6-high and -low expression groups (p=0.34; **Figure 7A**), the comprehensive genomic characterization revealed notable discrepancies in the genomic alteration landscape (**Figures 7B, C**). As shown in **Figure 7D**, *TP53*, *ARID1A*, and *FAT4* alterations were significantly enriched in the EPHB6-low expression group; whereas, *FGFR3*, *STAG2*, and *TENM3* alterations were more prevalent in the EPHB6-high expression group (p<0.05). Meanwhile, the specific DDR alteration landscape was also investigated; only *BRCA2* alterations (**Figure 7E**) were found to be significantly more frequent in the EPHB6-high expression group. When comparing alterations in the signaling pathway, alterations in RTK/RAS signaling were discovered to be more frequent in the EPHB6-high expression group, whereas alterations in TP53 signaling pathway were more prevalent in the EPHB6-low expression group (**Figure 7F**).

### 3.8 EPHB6 predicted molecular subtype and potential therapeutic strategies

**Figure 8:**
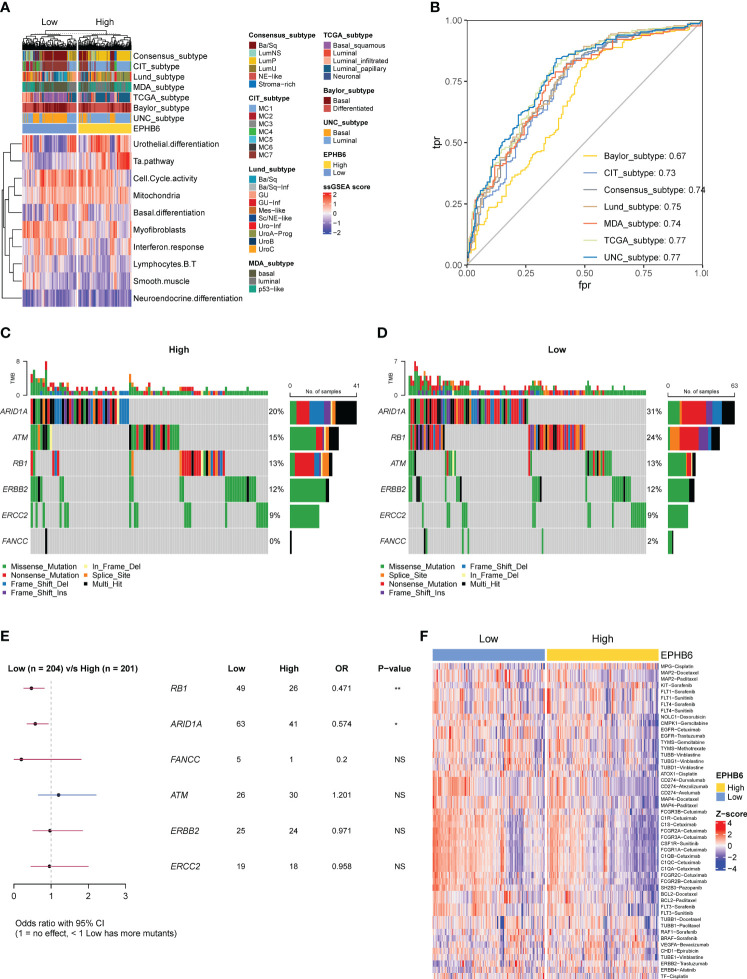
EPHB6 related molecular subtype and therapeutic strategies in bladder cancer. **(A)** Difference in the bladder cancer signatures between high- and low-EPHB6 group. **(B)** ROC curves showing the accuracy of EPHB6 for predicting molecular subtypes. **(C-E)** Difference in the prevalence of neoadjuvant chemotherapy-related genes between high- and low-EPHB6 group. **(F)** Difference in the enrichment scores of different therapeutic signatures between high- and low-EPHB6 group. Each therapeutic signature was indicated as target- regimen.

According to the seven molecular subtyping systems, a markedly higher proportion of luminal (papillary) subtype was observed to always occur in the EPHB6-high expression group; in comparison, patients with basal subtype were more abundantly enriched in the EPHB6-low expression group (**Figure 8A**). The enrichment scores of urothelial differentiation and Ta pathway were apparently higher in the EPHB6-high expression group, whereas the enrichment scores of myofibroblasts and IFN response seemed to be higher in the EPHB6-low expression group. Regarding cell cycle activity, mitochondria, basal differentiation, B and T lymphocytes, smooth muscle, and neuroendocrine differentiation, their enrichment scores between EPHB6-high and -low expression groups were nearly equivalent. Furthermore, the AUC values of EPHB6 predicting the molecular subtype were all > 0.70, except in the Baylor subtyping system (**Figure 8B**). These findings reveal that EPHB6 expression was highly correlated with molecular subtype accompanied with some specific molecular characteristics, which guided significance for the therapy selection to some extent. The chemotherapy-associated mutational profiling demonstrated that ARID1A (31% vs. 20%) and RB1 (24% vs. 13%) alterations were more prevalent in the EPHB6-low expression group (p<0.05; **Figures 8C-E**). In addition, the Drugbank database analysis demonstrated a significantly higher response to most chemotherapeutic and immunotherapeutic target drugs in the EPHB6-low expression group (**Figure 8F**).

### 3.9 Prognostic significance of EPHB6 in BLCA

**Figure 9:**
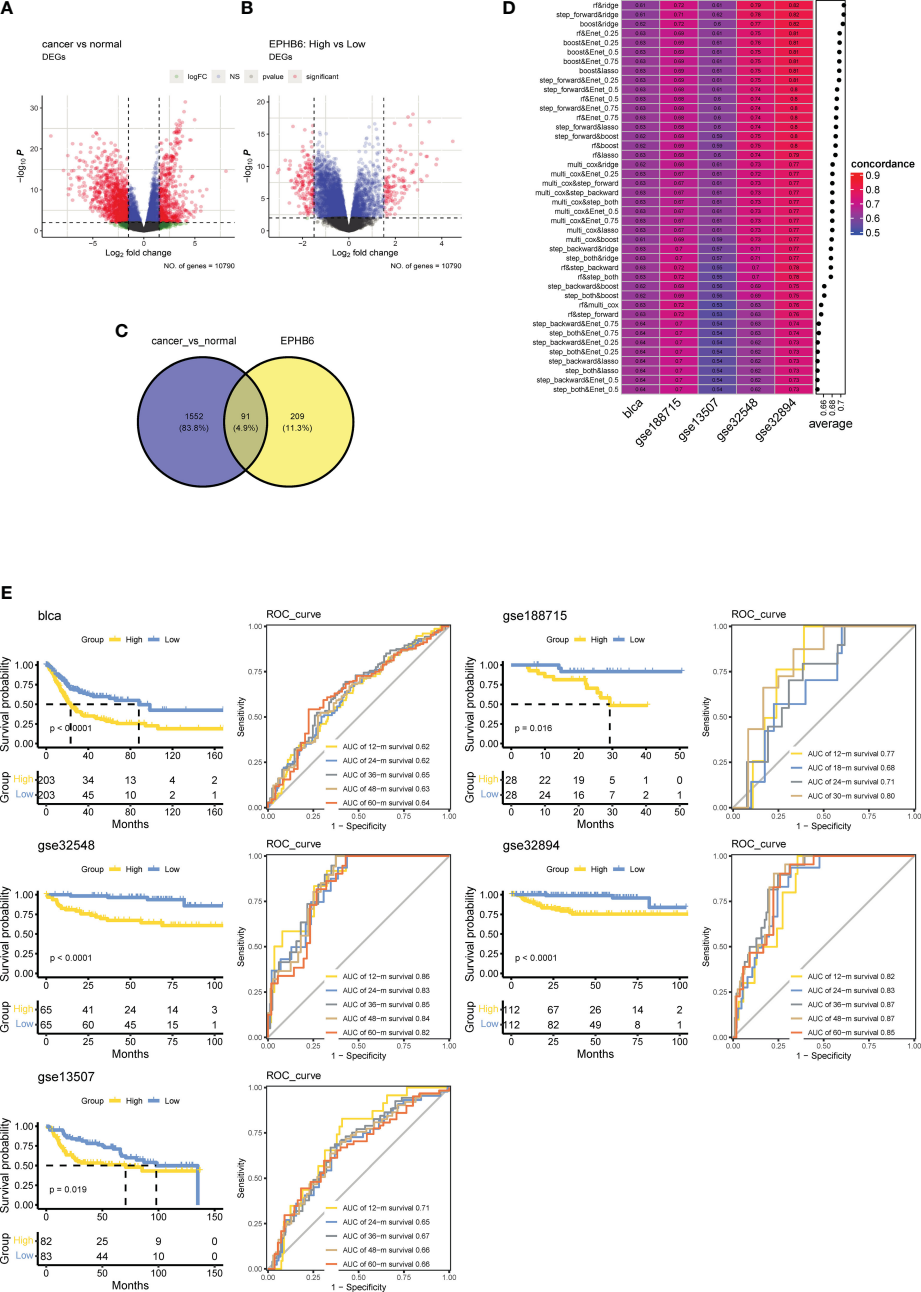
Establish a prognostic signature based on EPHB6 expression feature in bladder cancer. **(A)** Differentially expressed genes (DEGs) between tumor and normal bladder tissues as showing by the volcano plot. **(B)** Volcano plot showing the DEGs between high- and low-EPHB6 group. **(C)** Veen plot showing the shared genes. **(D)** Combination of machine learning algorithms for establishing EPHB6 related genes (EBGs) signatures. The C-index of each model was calculated across bladder cancer datasets, including TCGA-BLCA, GSE188715, GSE13507, GSE 32548 and GSE32894. **(E)** Kaplan-Meyer plots and time-dependent ROC curve for predicting survivals at 1- to 5-years in TCGA-BLCA, GSE188715, GSE13507, GSE 32548 and GSE32894 datasets.

Ninety-one overlapped genes between the DEGs of BLCA vs. normal tissues (**Figure 9A**) and EPHB6-related DEGs (**Figure 9B**) were acquired (**Figure 9C**; **Supplementary Table 2**). The univariate Cox regression analysis found 35 genes to be significantly associated with the prognosis of BLCA (**Supplementary Table 2**). After calculation, the combinational algorithms of RF and Ridge exhibited the best performance (**Figure 9D**), identifying a total of 21 genes enrolled in the EPHB6-related Genes signature (EBGs; **Supplementary Table 2**). According to the median cut-off value of EBGs score, patients with BLCA were divided into high- and low-EBGs score groups. In the TCGA-BLCA cohort, high EBGs score group had worse clinical outcome. Moreover, the performance of EBGs signature was validated in other four independent BLCA cohorts, and consistently higher EBGs score indicated an inferior OS. All the AUC values of EBGs signature in predicting prognosis for patients with BLCA were approximately 0.65 (**Figure 9E**).

## 4 Discussions

Globally, BLCA is one of the most prevalent malignant urinary carcinomas with approximately 550,000 new cases diagnosed and 170,000 deaths annually (45). Generally, BLCA can develop to NMIBC and MIBC tumors, and each subtype has specific clinicopathological and molecular features (46). Currently, surgical resection remains the major therapeutic strategy for the localized BLCA (47). For patients with NMIBC, intravesical therapies, such as Bacillus Calmette-Guérin or alternative therapies after resection are frequently used to prevent disease progression and recurrence. More aggressive therapies, including radical cystectomy and urinary diversion with chemotherapy, radiotherapy, neoadjuvant chemotherapy, or immunotherapy are mainly proposed for patients with MIBC (48). Despite that, patients with advanced or metastatic BLCA still have a poor prognosis, with an estimated 5-year survival rate of < 30%, especially with the difficulty of distinguishing patients who will and will not respond to therapy. Therefore, it is urgent to discover more novel biomarkers that can become effective therapeutic targets for BLCA.

In this study, we conducted the first pan-cancer analysis revealing the correlation between the largest family of RTKs and immunomodulation. Eph receptor/EFN ligand family genes expression was highly correlated with immunomodulation of TME in 32 types of cancer, and different Eph receptor/EFN ligand genes had distinct immunological effects in different types of cancer. BLCA is an immunogenic tumor type (49), and an in-depth investigation of Eph receptor/EFN ligand family genes in BLCA identified that inactive kinase EPHB6 may be a potential therapeutic target. Evidence has revealed that EPHB6 could shape a cold immune microenvironment in BLCA. Furthermore, lower expression of EPHB6 in BLCA indicated a higher T cell inflamed score, which can efficiently quantify T cell inflamed microenvironment, and such a score was confirmed to positively correlate with the response to immune checkpoints blockade (33). Moreover, IMvigor210 cohort analysis showed that lower EPHB6 expression may assist in predicting the inflamed, IC2+, and TC2+ phenotypes, which has been confirmed to be markedly correlated with immunotherapy response of patients with UC treated with atezolizumab (anti-PD-L1 agent) (34). In addition, in the IMvigor210 cohort, the patients with UC showing a complete or partial response had a lower EPHB6 expression, directly indicating that anti-EPHB6 immunotherapy may be a novel and reliable therapeutic strategy and could greatly help to promote the response to immunotherapy and improve clinical outcomes for patients with BLCA. Notably, pan-cancer analysis showed that EPHB6 could not exert the similarly cold immune effects in most other types of cancer; hence, anti-EPHB6 immunotherapy might be suitable for patients with BLCA only, but it requires further clinical investigations.

Immunomodulators, cancer immunity, tumor immune cells infiltration, and immune checkpoints have been frequently used to evaluate the immunological status of TME (50). Most chemokines and paired receptors, particularly CXCL9, CXCL10, CXCL11, CXCL13, CCL3, CCR2, CCR3, and CXCR3, stimulate the recruitment of CD8+ T cells in different types of cancer in humans (29, 51–53). As reported, the downregulated chemokine/receptors in the EPHB6-high expression group may result in a reduced activity of anti-cancer immunity. In addition, downregulation of immunostimulators may also cause a cold immune microenvironment in the EPHB6-high expression group. Meanwhile, the reduced expression of MHC molecules would also attenuate the ability of antigen presentation and processing (29). Comprehensively, the complex functions and interactions of these immunomodulators could be integrated and reflected by seven major steps, directly representing the anti-cancer immunity to tumor cells (30). In the EPHB6-high expression group, the activities of steps 1, 4, and 7 in the cancer immunity cycle were weakened from the perspective of molecular functions, which implies that the activities of cancer cell antigen release and immune cells tracking tumor cells were restricted. Owing to the reduced level of immunomodulators, the infiltrating levels of several effector cells including macrophages, dendritic cells, NK cells, and CD8+ T cells were all expectedly decreased, which was also validated in our local BLCA cohort. Collectively, EPHB6 shaped a cold immune microenvironment in BLCA. Although no drugs have been directly targeted at EPHB6, related pathway-regulating drugs could potentially affect its expression and/or activity and then regulate tumor cell biology. *In vitro* analysis has shown that drugs that inhibit PI3K could reactivate EPHB6, thereby suppressing lung cell metastasis and proliferation (54). Moreover, enzalutamide, a widely applied anti-androgen therapy in advanced prostate cancer, has been found to suppress EPHB6 expression by binding to the androgen-response-element on the EPHB6 promoter to stimulate tumor cell metastasis (55). Therefore, given that EPHB6 has been identified as a protective prognostic factor in BLCA, monotherapy using its related targeted therapy could potentially lead to a worse clinical outcome. However, for patients with advanced or metastatic BLCA, EPHB6-directed therapy could potentially serve as a stimulator to immune checkpoint inhibitors (ICIs), reprogramming a cold immune tumor microenvironment into a hot one, which merited further study and investigation.

The comprehensive functional characterization subsequently displayed the enrichment of immunological processes, irrespective of initial (antimicrobial humoral immune response mediated by antimicrobial peptide) and adaptive immune responses, which further confirmed that EPHB6 expression was remarkably associated with immunomodulation in BLCA. Especially, IFN-γ (the only member belonging to the type II interferon family), as the uppermost cytokine involved in the anti-cancer immunity, may function to inhibit angiogenesis, induce apoptosis of cancer cells and T regulatory (T_reg_) cells, and further activate M1 macrophages to hinder tumor progression (56). However, some researchers have reported that IFN-γ may contribute to the development and progression of tumor (57–60). In this study, the findings revealed that IFN-γ signature enrichment score was markedly higher in the EPHB6-low expression group compared to that in the EPHB6-high group. The higher level of IFN-γ signaling likely contributed to the excessively inflamed microenvironment, consequently causing the inferior prognosis (OS, DSS, and PFI) in the EPHB6-low expression. From another perspective, IFN-γ is widely believed to be a critical factor influencing the response of immunotherapy (61). Ayers et al. has reported that patients with metastatic cancer (gastric cancer, head and neck squamous cell carcinoma, and melanoma) that responded to anti-PD-L1/PD-1 immunotherapy had a higher expression level of IFN-γ signaling-related genes (IFNG, CXCL9, CXCL10, HLA-DRA, STAT1, and IDO1) compared with the non-responders (33). A similar result was obtained in urothelial, melanoma, and non-small cell lung carcinoma in which IFN-γ signaling-related genes, including IFNG, CD274, LAG3, and CXCL9, could well predict about who could benefit from the immunotherapy *via* anti-PD-L1/PD-1 antibody (62, 63). Mechanistically, IFN-γ could activate the integrative inflammatory response and related immunological signaling to achieve the enhanced immunotherapy efficiency. Moreover, EPHB6 expression was negatively correlated with the enrichment scores of immunotherapy-predicted pathways.

Remarkably, mutational spectrum analysis further showed that the EPHB6-low expression group had a higher enrichment of *TP53* genomic alterations, whereas especially *FGFR3* alterations were abundantly enriched in the EPHB6-high expression group. Altered *TP53* is known to prevalently associate with the poor clinical outcome in BLCA, and generally, *TP53* alteration frequently occurred in patients with advanced or metastatic BLCA (64). In contrast, *FGFR3* alterations were significantly correlated with lower pT stage, lower tumor grade, and expectedly longer survival in BLCA (65). From these molecular characteristics, EPHB6 was verified to be a potential prognostic biomarker. Additionally, EPHB6 expression may help identify the patients with altered *FGFR3*. Erdafitinib, as a later-line therapeutic regimen targeting FGFR3, recently achieved great advances in treating patients with UC having *FGFR3* alterations (66). In addition, *FGFR3*-altered BLCA tumors had a lower expression of a fibroblast TGF-β response signature and downregulation of epithelial–mesenchymal transition signature (67). Accordingly, higher expression of EPHB6 was associated with the lower immune and stromal score in both TCGA-BLCA and local BLCA cohorts, and two other independent BLCA datasets. *TP53* alterations were found to be positively correlated with the immune-promoting microenvironment; however, *FGFR3* alterations might contribute to the cold immune microenvironment in BLCA. Furthermore, *FGFR3* alterations were prevalent in the luminal papillary MIBC subtype (68); as expected, the EPHB6-high group had the higher proportion of luminal (papillary) subtype. Based on the molecular subtype classification, the EPHB6-low group showed a notable enrichment of basal subtype. Correspondingly, the enrichment scores of urothelial differentiation and Ta pathway were observed to be higher in the EPHB6-high expression group. As reported, urothelial differentiation is upregulated in the luminal subtype (69), and Ta pathway is positively correlated with FGFR3 alterations (70). Therefore, not only does EPHB6 have the prediction ability in prognosis in BLCA but also in molecular subtype.

At the molecular level, different molecular subtypes were noted to be correlated with distinct responses to chemotherapy, radiotherapy, neoadjuvant chemotherapy, and immunotherapy (71–74). A previous study regarding the consensus subtype of MIBC reported that receiving immunotherapy was more suitable for basal subtype tumors (38). Moreover, PURE-01 (NCT02736266) study also demonstrated that basal type tumors had the highest infiltrating level of tumor immune cells and pathological response rate to the neoadjuvant pembrolizumab (75). As previously described, *RB1* (76) and *ARID1A* (77) alterations highly correlated with the response of chemotherapy. Both *RB1* and *ARID1A* alterations were prevalently altered in the EPHB6-low expression (enriched basal subtype) group suggesting that patients with low expression of EPHB6 were more likely to be sensitive to the chemotherapeutic drugs. The Drugbank database analysis predicted that the EPHB6-low expression group had the higher response to most chemotherapeutic and immunotherapeutic regimens. Overall, adjuvant or neoadjuvant chemotherapy and immunotherapy (either monotherapy or combination therapy) could be considered for patients with BLCA having low expression of EPHB6. Based on the EPHB6-determined transcriptomic profile, a novel EBGs signature was eventually established which performed more robustly in the prognosis prediction.

In this study, the role of Eph receptor/EFN ligand family genes was investigated in a pan-cancer cohort for the first time. EPHB6 exhibited unique cold immune effects on BLCA tumors. From multiple perspectives, it was established that anti-EPHB6 immunotherapy might be a novel therapeutic regimen for patients with BLCA, which would greatly improve the clinical outcomes of BLCA. However, there are some limitations in the present study. First, number of patients in our local BLCA cohort was small; therefore, it is necessary to validate these results in a larger cohort of patients with BLCA. Second, the performance of EPHB6 in predicting the response of immunotherapy was only verified in a IMvigor210 cohort, which should be further investigated. Third, experimental explorations and clinical trials should be proposed to testify the efficacy and efficiency of anti-EPHB6 immunotherapy.

## Data availability statement

The raw data supporting the conclusions of this article will be made available by the authors, without undue reservation.

## Ethics statement

The studies involving human participants were reviewed and approved by The ethics committee of Ningbo First Hospital. The patients/participants provided their written informed consent to participate in this study.

## Author contributions

XJ, JJ, and LX contributed to conception and design of the study. DZ and CZ organized the database. DZ performed the statistical analysis. XJ wrote the first draft of the manuscript. ZY, and ZJ wrote sections of the manuscript. XJ, DZ, CZ contributed equally to this work. All authors contributed to manuscript revision, read, and approved the submitted version.

## Supplementary material

Supplementary Figure 1 Correlation matrix showing the relationship between the expression levels of all the Eph receptors and EFN ligands and immunomodulatory genes a in pan-cancers.

Supplementary Figure 2 EPHB6 is associated with a cold immune microenvironment in Bladder cancer. **(A)** Heatmap showing the elevated in the expression level of marker genes of CD8 T cells, dendric cells, macrophages, NK cells and Th1 cells. **(B)** Correlation between the expression levels of immune checkpoints and EPHB6 in TCGA-BLCA cohort.

Supplementary Figure 3 Difference in the expression levels of immunomodulatory genes between the high- and low- EPHB6 groups in GSE31684 dataset.

Supplementary Figure 4 Correlation analysis of the mRNA expression level of EPHB6 and CD8A or CD68 in TCGA-BLCA, Imvigor210 and Local BLCA datasets.

Supplementary Figure 5 Binary response stratified in IMvigor210 cohort by *TP53* mutation (mt vs wt) and EPHB6 expression level (high vs low). Mt: mutated; wt: wildtype.
