# Supplementary material for: Eph receptor B6 shapes a cold immune microenvironment, inhibiting anti-cancer immunity and immunotherapy response in bladder cancer
Source: Front Oncol. 2023 Aug 9;13:1175183. doi: 10.3389/fonc.2023.1175183 (PMC10450340; doi:10.3389/fonc.2023.1175183)

Supplementary Figure 1

A

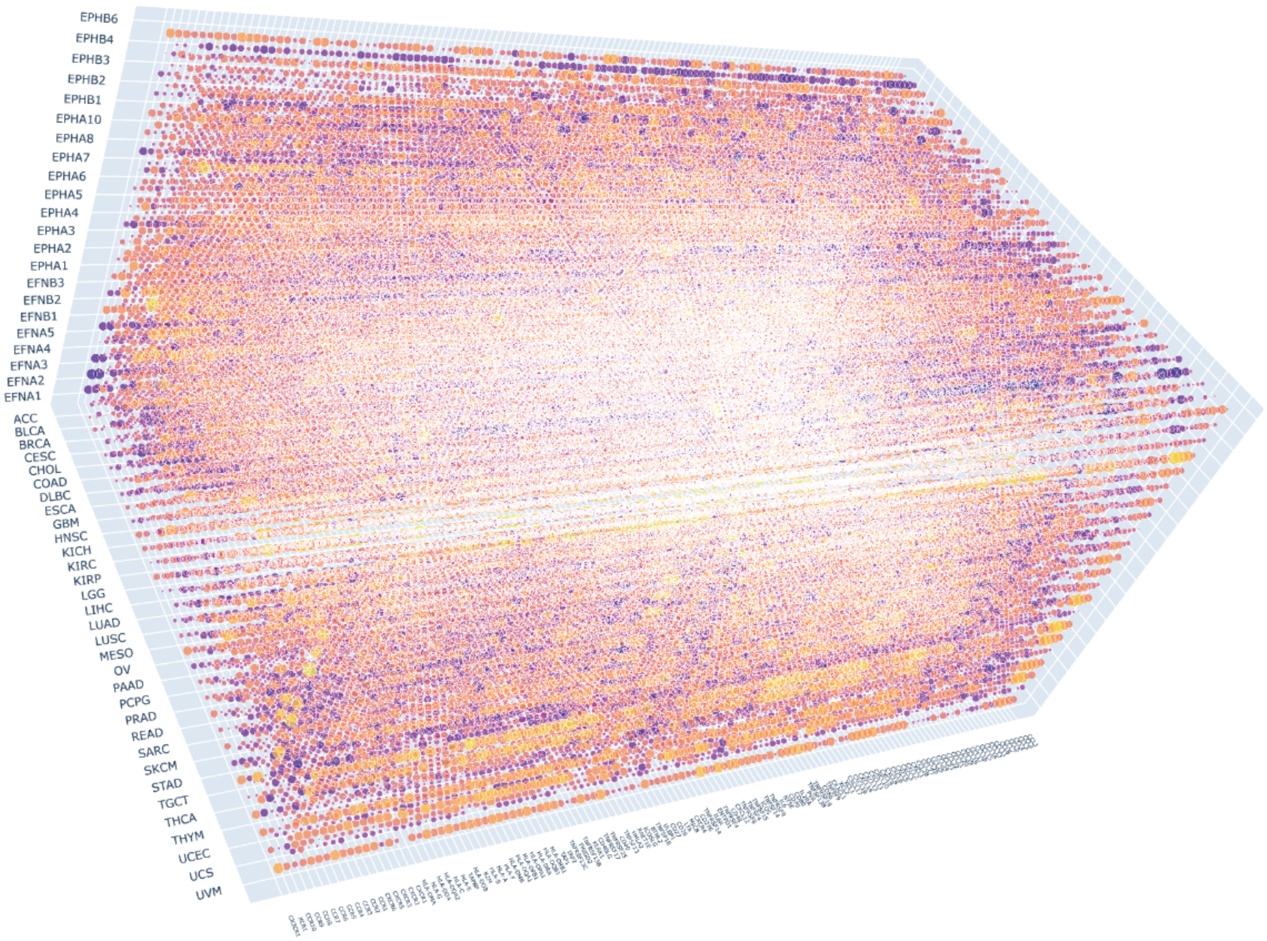

B

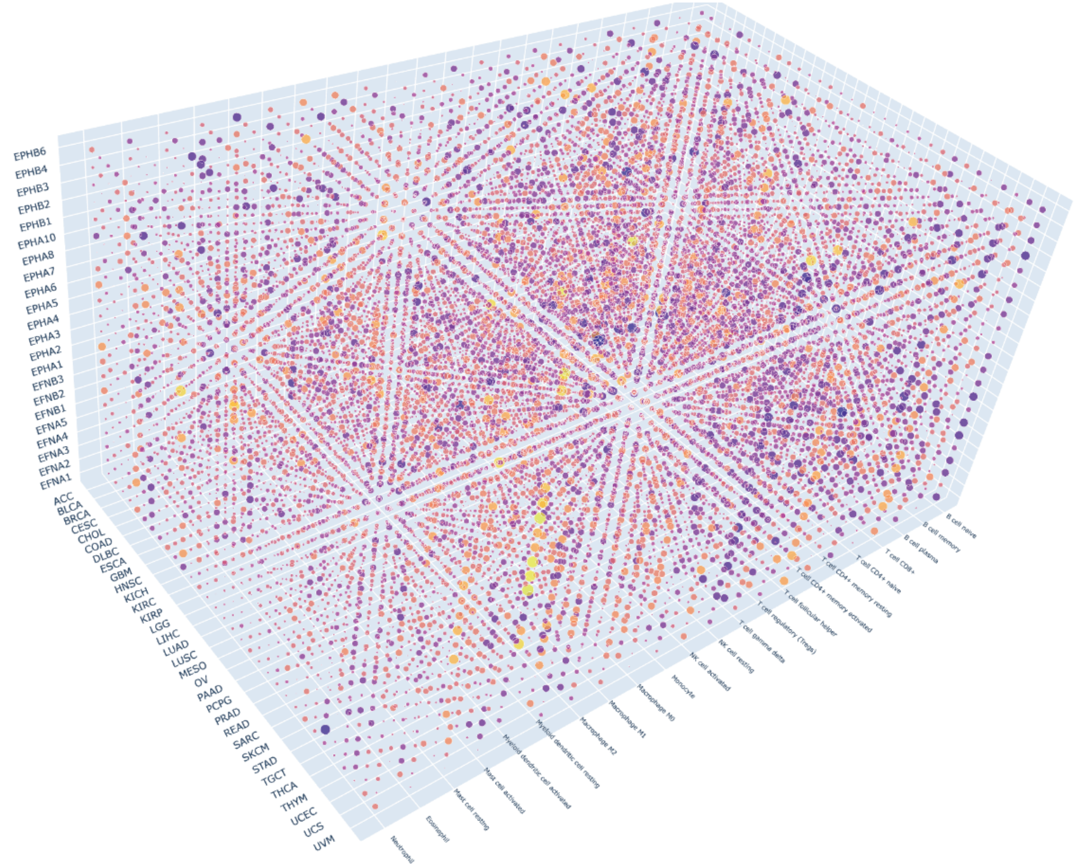

Supplement: Supplementary Figure 1 — Correlation matrix showing the relationship between the expression levels of all the Eph receptors and EFN ligands and immunomodulatory genes a in pan-cancers. [file DataSheet_1.pdf]
