# Supplementary figures and images for: Eph receptor B6 shapes a cold immune microenvironment, inhibiting anti-cancer immunity and immunotherapy response in bladder cancer
Source: Front Oncol. 2023 Aug 9;13:1175183. doi: 10.3389/fonc.2023.1175183 (PMC10450340; doi:10.3389/fonc.2023.1175183)

A

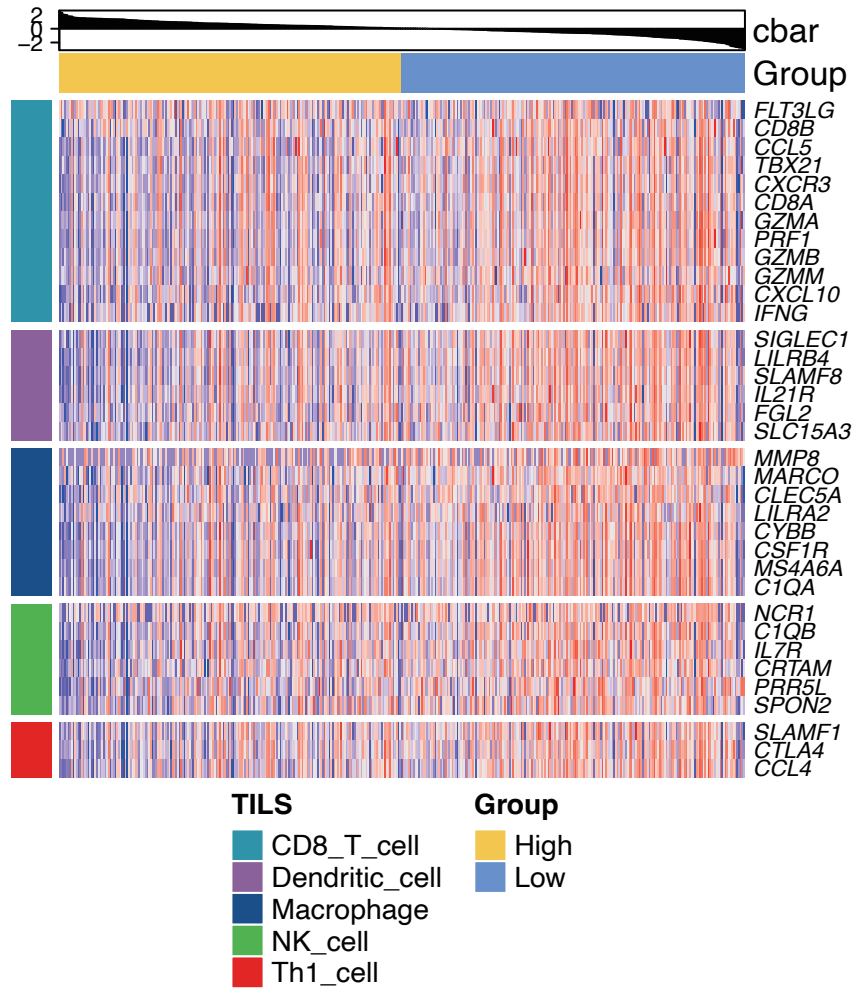

B

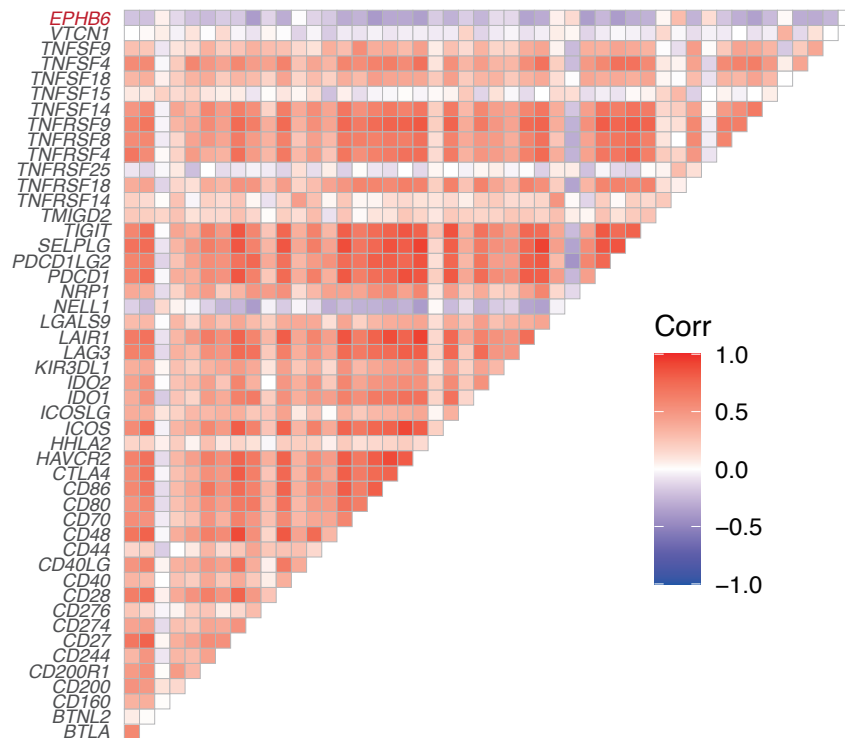

Supplement: Supplementary Figure 2 — EPHB6 is associated with a cold immune microenvironment in Bladder cancer. (A) Heatmap showing the elevated in the expression level of marker genes of CD8 T cells, dendric cells, macrophages, NK cells and Th1 cells. (B) Correlation between the expression levels of immune checkpoints and EPHB6 in TCGA-BLCA cohort. [file DataSheet_2.pdf]

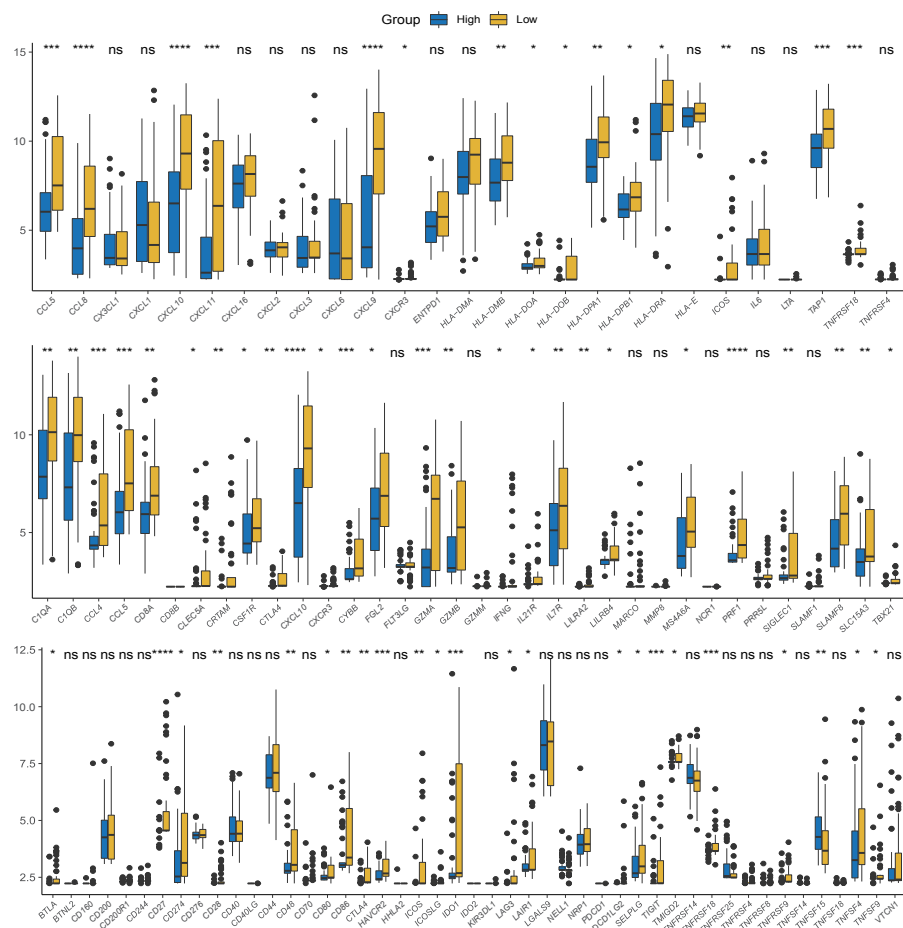

Supplement: Supplementary Figure 3 — Difference in the expression levels of immunomodulatory genes between the high- and low- EPHB6 groups in GSE31684 dataset. [file DataSheet_3.pdf]

## TCGA\_BLCA

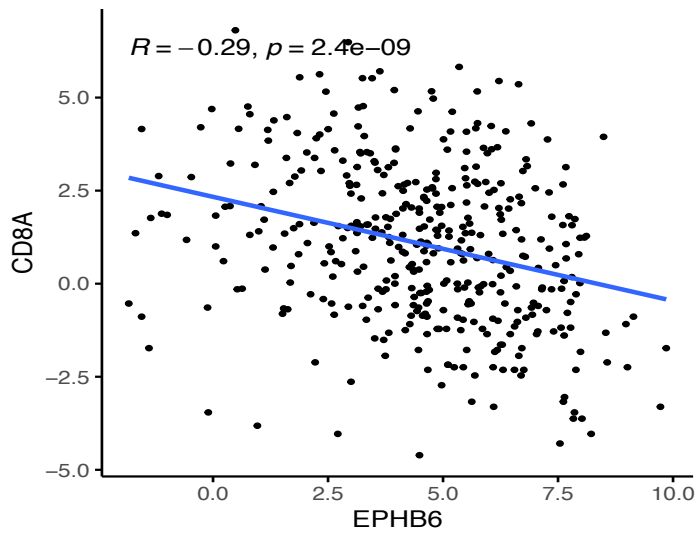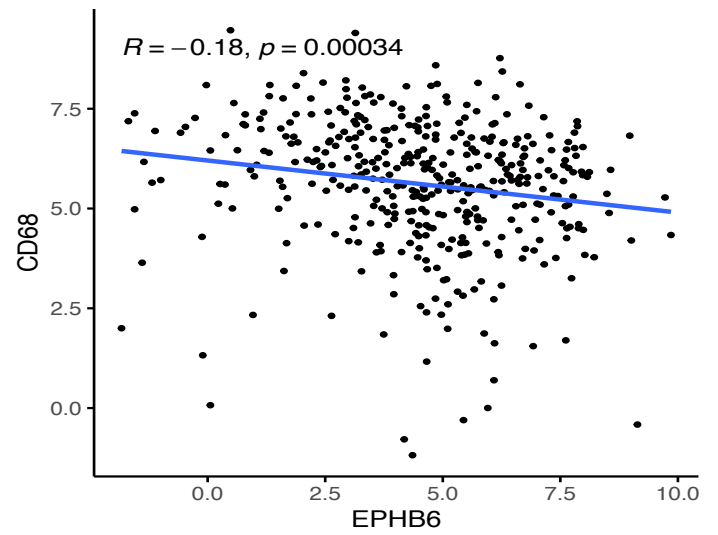

## Imvigor210

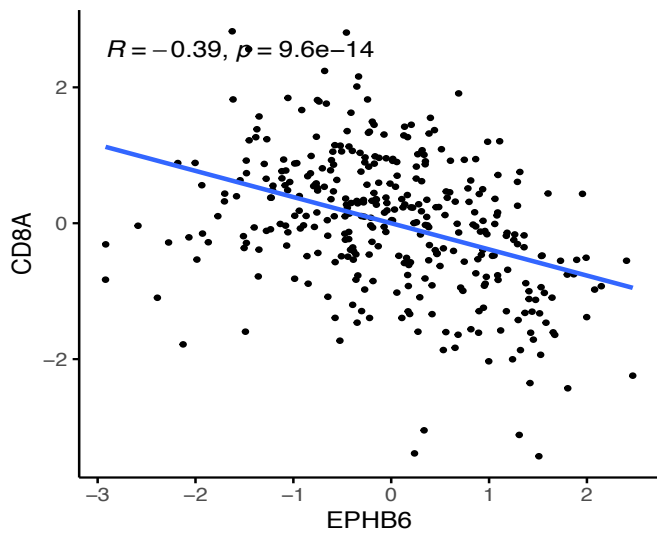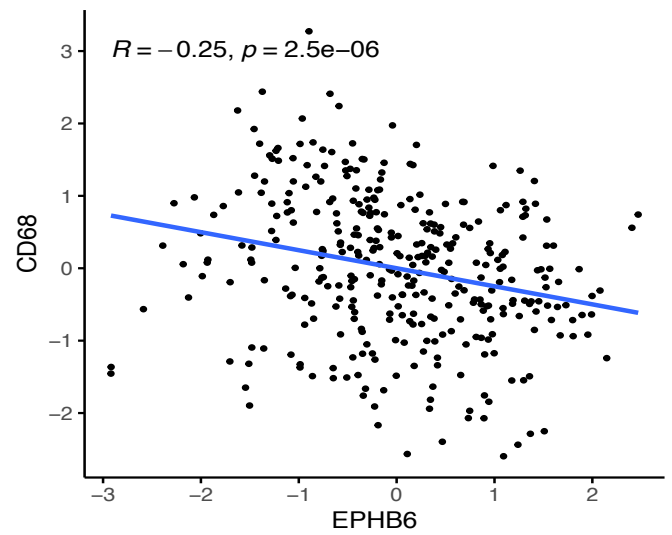

## Local

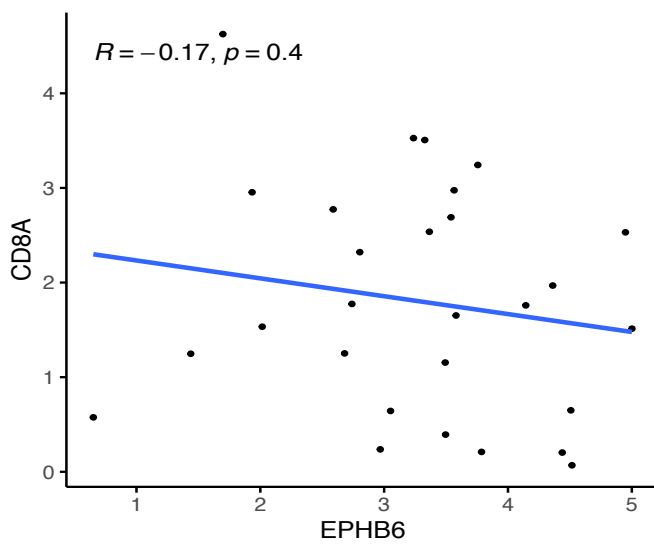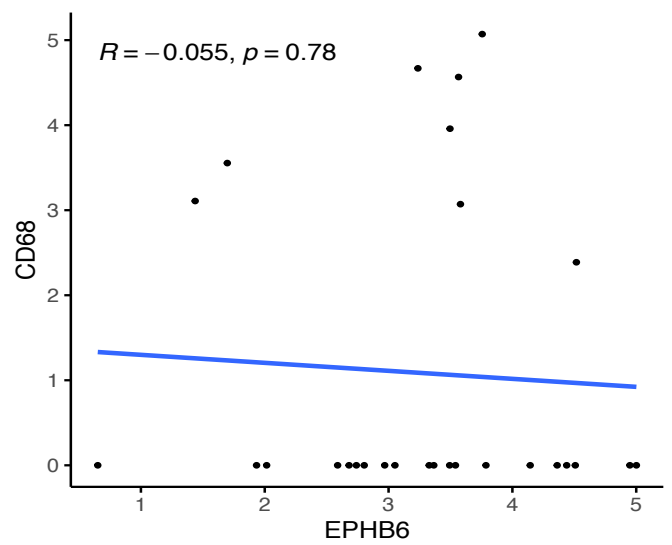

Supplement: Supplementary Figure 4 — Correlation analysis of the mRNA expression level of EPHB6 and CD8A or CD68 in TCGA-BLCA, Imvigor210 and Local BLCA datasets. [file DataSheet_4.pdf]

# TP53 status + EPHB6

CR/PR SD/PD

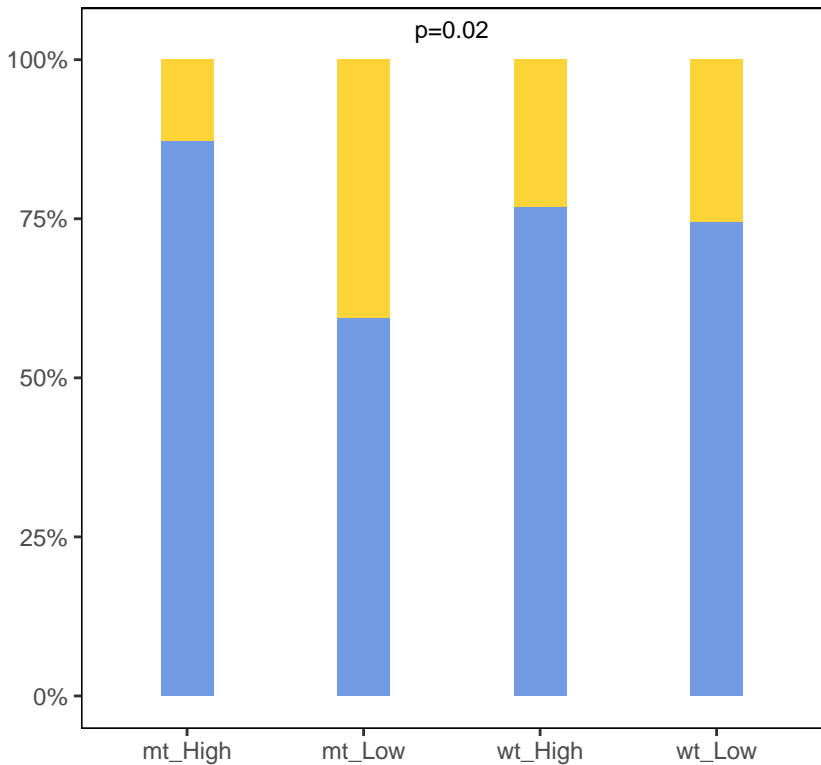

Supplement: Supplementary Figure 5 — Binary response stratified in IMvigor210 cohort by TP53 mutation (mt vs wt) and EPHB6 expression level (high vs low). Mt: mutated; wt: wildtype. [file DataSheet_5.pdf]
